# Trust in Information Sources and COVID‐19 Vaccine Uptake

**DOI:** 10.1002/puh2.70145

**Published:** 2025-10-28

**Authors:** Peijia Zha, Rubab Qureshi, Ganga Mahat, Le Gao, Catherine Garcia, Zhi Wei

**Affiliations:** ^1^ Division of Nursing Science School of Nursing Rutgers the State University of New Jersey Newark New Jersey USA; ^2^ Division of Entry to Baccalaureate Nursing School of Nursing Rutgers the State University of New Jersey Newark New Jersey USA; ^3^ Department of Computer Science New Jersey Institute of Technology Newark New Jersey USA

**Keywords:** COVID‐19 vaccine uptake, information sources, mistrust, trust

## Abstract

**Introduction:**

The COVID‐19 pandemic highlighted the important role of trust in information sources in shaping vaccine uptake behavior. This study aimed to examine the relationship between trust in various information sources and COVID‐19 vaccine uptake.

**Methods:**

Data were analyzed from the April 2021 wave of the Health Reform Monitoring Survey (HRMS). A total of 9067 US adults were included. Multivariate logistic regression models were used to assess associations between seven different information sources, trust, and vaccine uptake, adjusting for sociodemographic factors.

**Results:**

Trust in personal doctors/healthcare providers was the strongest predictor of COVID‐19 vaccine uptake (adjusted odds ratio [AOR] = 1.53, *p* < 0.001), followed by trust in other doctors/community healthcare providers (AOR = 1.40, *p* < 0.001) and pharmacists (AOR = 1.32, *p* < 0.001). Non‐healthcare information sources, such as social service, neighborhood, or civic organizations (ARO = 1.27, *p* < 0.001), and local elected officials (AOR = 1.08, *p* < 0.001), showed weaker associations. Trust in religious leaders and family/friends was associated with a lower likelihood of vaccine uptake (AOR = 0.81, *p* < 0.001, and AOR = 0.85, *p* < 0.001, respectively). Older age, higher education, and Hispanic ethnicity were positively linked to COVID‐19 vaccine uptake.

**Conclusion:**

Trust in healthcare professionals significantly influences COVID‐19 vaccine uptake, whereas trust in non‐healthcare sources plays a more limited role. Religious leaders’ and family/friends’ influence was minimal. Building and maintaining trust in healthcare providers is crucial for promoting vaccine acceptance, particularly amid widespread misinformation and political polarization. Public health efforts should prioritize improving healthcare provider–patient communication, utilizing trusted healthcare figures as role models, and implementing culturally tailored interventions to address hesitancy among marginalized groups.

## Graphical abstract

Trust in healthcare providers was the strongest predictor of COVID‐19 vaccine uptake, whereas reliance on religious leaders and family/friends was associated to lower vaccine uptake. Findings of this study demonstrate the crucial role of healthcare professionals in fostering vaccine acceptance amid misinformation and political polarization.

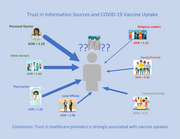

## 1 Introduction

Vaccines have been one of the most effective public health tools in reducing morbidity and mortality associated with preventable infectious diseases. The COVID‐19 vaccines served as an important public health tool in containing the pandemic, and their efficacy has been extensively confirmed by science. According to Thompson et al. [1], the estimated efficacy of the mRNA COVID‐19 vaccine in preventing infection was 90% with full immunization (two doses of any 2024–2025 COVID‐19 vaccine 6 months apart) and 80% with partial immunization. These results suggested that approved mRNA COVID‐19 vaccinations are useful in preventing SARS‐CoV‐2 infection. As of August 2024, 70% of the world's population has received more than 13 billion doses of the COVID‐19 vaccines [2]. In the United States, approximately 270.2 million (81%) had received at least one COVID‐19 vaccine dose, and 230.6 million (70%) were fully vaccinated by 2023 [2]. An estimated 984.4 million doses were distributed nationwide and were made available free of cost, yet only 676.7 million doses (68.74%) had been administered. A search of the published literature on the low uptake of the COVID‐19 vaccine yielded thousands of references to multiple barriers that predicted vaccine hesitancy. The barriers could be structural, systemic, and/or individual factors. One individual factor is trust: trust in healthcare systems, trust in healthcare providers, trust in the government, and trust in the COVID‐19 vaccine. Trust is often dependent on factors rooted in local social, cultural, structural, systemic, and individual characteristics and experiences and is associated with vaccine uptake and hesitancy [3]. Although several studies reported on trust in healthcare systems, healthcare providers, governments, and the vaccine itself, where people get their information about vaccines and what sources are most trusted are also important predictors of vaccine uptake and vaccine hesitancy.

Trust in information sources plays an important role in shaping health behaviors, including vaccine uptake. Individuals often rely on trusted sources to weigh the risks and benefits of vaccination, especially when faced with limited or conflicting information [4]. The role of trust in information sources is further complicated by the diversity of available channels. During the COVID‐19 pandemic, individuals accessed information from various sources, including community numbers (including healthcare providers), researchers, media organizations, social media, and government officials [37]. Healthcare providers are generally viewed as the most trusted source of vaccine information [5, 6]. Research suggests that trust in healthcare providers and instructions positively correlates with vaccine acceptance, whereas mistrust can lead to vaccine hesitancy [7]. Conversely, trust in unofficial sources, such as social media and social networks, can sometimes negatively impact uptake, especially when misinformation is prevalent [7, 8]. This is particularly important in the context of COVID‐19, where the novelty of the virus and the rapid development of vaccines created a heightened sense of vulnerability [9].

Trust in government officials and social media influencers varies widely, often along political and ideological lines. COVID‐19 presented a whole new wave of anti‐vaccination sentiment in the United States, turning a seemingly small subculture into a strong movement that grew in popularity [10]. With the pandemic occurring during a heavily polarized political climate, then President Donald Trump's remarks on COVID‐19 being a “hoax,” downplaying the seriousness of the virus, and undermining precautions [11] further enabled anti‐vaccine sentiment to align with conservative ideologies about medical freedoms [10]. Government officials have used populist ideas of putting “the common people” against “expert elites” to undermine public health officials’ warnings and advice [12], such as vaccine mandates, which have historically been shown to garner public resistance against government‐mandated vaccination programs [13]. Anti‐vaccine sentiment has also been spread via influencers [10], who are people who have a large internet presence and a large audience or following. An influencer may be more believable at face value if they have built a good rapport with their audience [10]. These variabilities highlight the need for targeted public health strategies that use trusted information sources to counter misinformation and promote vaccine uptake.

Misinformation, such as false claims that “vaccines cause infertility,” leads to risk perception bias in certain populations [14]. In the United States, political polarization is evident in vaccine uptake. Republican supporters were less willing to be vaccinated than Democrats [15]. Furthermore, racial minorities have long been skeptical of vaccines due to historical medical exploitation incidents, such as the Tuskegee syphilis experiment [16]. In addition, public confidence in COVID‐19 vaccine faced further strain as variants like omicron and delta reduced vaccine effectiveness against infection [17]. These challenges highlight the urgent need to understand how “trust in information sources” from healthcare providers, government, and community affects vaccine uptake behavior. Addressing this “trust gap” could inform strategies to combat hesitancy and improve global vaccine equity.

This study aimed to explore the relationship between trust in various information sources, such as healthcare providers, religious leaders, neighborhoods, and family/friends, and the likelihood of COVID‐19 vaccine uptake among US adults. Specifically, we hypothesize that (1) higher trust in healthcare information sources (personal doctors, other doctors, and pharmacists) will be associated with a greater likelihood of COVID‐19 vaccine uptake and (2) higher trust in non‐healthcare information sources (local elected officials, social service, neighborhood, or civic organizations, religious leaders, and family/friends) will demonstrate weaker or more variable associations with vaccine uptake compared to healthcare sources. By understanding which sources most strongly influence vaccine decisions across populations, this study seeks to inform actionable public health communication strategies to address hesitancy and improve vaccination rates.

## 2 Methods

This study analyzed data from the Health Reform Monitoring Survey (HRMS), an internet‐based survey launched in January 2013 by the Urban Institute in the United States. The HRMS provides early insights into health‐related behaviors and attitudes, particularly among the nonelderly population (ages 18–64). The April 2021 wave of the HRMS included nine key areas: self‐reported health status, health insurance coverage, access to healthcare, awareness of marketplace and Medicaid coverage options, use of public benefits, telehealth usage, COVID‐19 vaccine attitudes, forgone care due to the COVID‐19 pandemic, and unfair treatment in healthcare settings. Among these key areas, questions on COVID‐19 vaccine attitudes, trust in information sources, and vaccine uptake were included. The study analyzed the responses from 9067 participants regarding COVID‐19 vaccine uptake and trust in seven information sources. Key demographic information included age, gender, race/ethnicity, education level, income, and employment status.

### 2.1 Measures

Outcome variable: COVID‐19 vaccine uptake, the outcome variable, was assessed by the question, “Have you received at least one dose of a COVID‐19 vaccine?” Responses were coded as yes (1) and no (0) for analysis.

Explanatory variables: Trust in information sources was measured through participants’ ratings of seven distinct source types on a 5‐point Likert scale ranging from “strongly distrust” (1) to “strongly trust” (5). The sources included (1) personal doctors or healthcare providers, (2) other doctors or healthcare providers in the community, (3) pharmacists, (4) religious leaders, (5) local elected officials, (6) social service, neighborhood, or civic organizations, and (7) family and friends.

Sociodemographic variables: The analysis adjusted for several key sociodemographic variables known to affect vaccine uptake, including age, education, race/ethnicity, health insurance coverage, and self‐reported health status. These variables were included as covariates in all adjusted models to account for potential confounding. The inclusion of self‐reported health status is particularly relevant given its established association with both health behaviors and trust in information sources.

### 2.2 Data Analysis

All data analyses were conducted by R 4.3.1 with the multivariate imputation by chained equations (MICE) package for Windows. Data were downloaded from the Inter‐University Consortium for Political and Social Research and the Regents of the University of Michigan. All study variables were systematically checked for special values, including “refused” and “not asked,” which were recoded as missing data. Additionally, given one key explanatory variable, trust in “other doctors or healthcare providers in your community” had 32% of missing data. MICE was performed to create a complete dataset for analysis, avoiding the potential bias and loss of power from listwise deletion [18].

Descriptive statistics were used to summarize the characteristics of the study population. Bivariate *χ* ^2^ tests and independent *t*‐test were conducted to assess the associations between COVID‐19 vaccine uptake, trust in information sources about COVID‐19 vaccines, and sociodemographic factors of participants. In order to address the ordinal nature of the 5‐point Likert scale trust variables, a two‐step diagnostic process was applied. First, a full logistic regression model was fitted with the trust in information sources variables treated as ordered predictors, allowing for tests of nonlinear effects. As the nonlinear components were not statistically significant, a parsimonious final model was adopted, treating trust in information sources variables as monotonic predictors under the assumption of a consistent linear trend. The final analysis consisted of a single multivariable logistic regression model assessing the probability of vaccine uptake as a function of explanatory variables. Specifically, this model included seven trusts in information sources variables simultaneously. This approach allowed for the estimation of the unique effect of each trust in the information source, while controlling for the influence of the other study variables.

## 3 Results

Among the April 2021 wave of the HRMS (*n* = 9067), the median age of participants was 46 years old (interquartile range [IQR] = 34–56 years). Forty‐nine percent (*n* = 4476) of participants were female, and 50.63% were male (*n* = 4591). Sixty‐three percent of the participants identified as White (*n* = 5715), and 18.1% were Hispanic (*n* = 1639). More than a third of participants reported having a bachelor's degree or higher education (*n* = 3272). More than half, 55.61% (*n* = 5024) of participants reported they did not receive at least one dose of a COVID‐19 vaccine. Thirty‐seven percent (*n* = 3375) of participants reported they had good health. Detailed sample characteristics are presented in Table 1.

**TABLE 1:** Demographic characteristics of study sample (*N* = 9067).

	*N*	%
Gender		
Male	4591	50.63
Female	4476	49.36
Education		
No high school diploma or GED	596	6.57
High school graduate	2161	23.83
Some college or associate's degree	3038	33.51
Bachelor's degree or higher	3272	36.09
Race/Ethnicity		
White, non‐Hispanic	5715	63.03
Black, non‐Hispanic	987	10.89
Other, non‐Hispanic	396	4.37
2 more races, non‐Hispanic	330	3.64
Hispanic	1639	18.08
Self‐reported health		
Excellent	905	9.98
Very good	3227	35.59
Good	3375	37.22
Fair	1284	14.16
Poor	255	2.81
COVID‐19 vaccine uptake (at least one dose)		
Yes	3974	43.83
No	5042	55.61

*Note:* Percentages may not add to 100 due to missing data and rounding.

Bivariate analyses revealed that participants who reported receiving the COVID‐19 vaccine were significantly older than those who did not (*t* = −17.85, *p* < 0.001). Vaccine uptake also differed by education level (*χ* ^2^ = 380.57, *p* < 0.001), with a greater proportion of vaccinated participants holding a bachelor's degree or higher compared to those unvaccinated (46.6% vs. 27.9%). Significant differences were revealed by race/ethnicity (*χ* ^2^ = 28.52, *p* < 0.001), with non‐Hispanic White participants more likely to be vaccinated. General health status was also significantly associated with vaccine uptake (*χ* ^2^ = 33.98, *p* < 0.001); vaccinated participants more frequently reported “very good” health, whereas unvaccinated participants were more likely to report “good” health. See Table 2 for details.

**TABLE 2:** Sociodemographic characteristics of participants by COVID‐19 vaccine uptake status.

		COVID‐19 vaccine uptake (yes)		COVID‐19 vaccine uptake (no)			
		Mean	SD	Mean	SD	*t*	*p*
Age		47.46	12.58	42.69	12.65	−17.85	<0.001

		*N*	%	*n*	%	*χ* ^2^	*p*
Education	No high school or GED	184	4.63	408	8.09	380.57	<0.001
	High school or GED	703	17.69	1448	28.72		
	Some college or associate's degree	1235	31.08	1779	35.28		
	Bachelor's degree or higher	1852	46.60	1407	27.91		
Race/Ethnicity	White, non‐Hispanic	2555	64.29	3135	62.18	28.52	<0.001
	Black, non‐Hispanic	375	9.44	600	11.90		
	Other, non‐Hispanic	209	5.26	185	3.67		
	2+ races, non‐Hispanic	138	3.47	189	3.75		
	Hispanic	679	17.54	933	18.50		
General health	Excellent	377	9.51	525	10.44	33.98	<0.001
	Very good	1517	38.26	1693	33.66		
	Good	1478	37.28	1876	37.30		
	Fair	503	12.69	771	15.33		
	Poor	90	2.27	165	3.28		

Trust in information sources about the COVID‐19 vaccine varied significantly between vaccinated and unvaccinated participants across all seven sources examined (*p* < 0.001). Vaccinated participants reported substantially higher levels of trust in healthcare information sources, including their personal doctors (66.62% vs. 33.55%), other healthcare providers (49.02% vs. 22.10%), and pharmacists (40.03% vs. 18.54%), on “strongly trust.” By contrast, unvaccinated participants were more likely to report distrust toward these sources. Trust in religious leaders, elected officials, social service, neighborhood, or civic organizations, and family and friends also showed significant differences, although patterns were less pronounced. Overall, vaccinated individuals consistently demonstrated higher trust in healthcare professionals, whereas unvaccinated participants were more likely to express distrust across other information sources (see Table 3).

**TABLE 3:** Trust in information sources for COVID‐19 vaccine by vaccine uptake status.

How much trust in following source for information about a COVID‐19 vaccine		COVID‐19 vaccine uptake (yes)		COVID‐19 vaccine uptake (no)			
		*n*	%	*n*	%	*χ* ^2^	*p*
Your personal doctor/Your personal healthcare provider/A doctor or healthcare provider in your community	Strongly trust	2639	66.62	1678	33.55	1200.98	<0.001
	Somewhat trust	1076	27.16	1860	37.19		
	Neither trust or distrust	219	5.53	1076	21.52		
	Somewhat distrust	21	0.53	190	3.80		
	Strongly distrust	6	0.15	197	3.94		
Other doctors or healthcare providers in your community	Strongly trust	1504	49.02	683	22.10	748.33	<0.001
	Somewhat trust	1238	40.35	1304	42.19		
	Neither trust or distrust	288	9.39	872	28.21		
	Somewhat distrust	32	1.04	131	4.24		
	Strongly distrust	6	0.20	101	3.27		
Pharmacists in your community	Strongly trust	1584	40.03	925	18.54	1017.41	<0.001
	Somewhat trust	1748	44.17	1837	36.82		
	Neither trust or distrust	566	14.30	1685	33.77		
	Somewhat distrust	43	1.09	284	5.69		
	Strongly distrust	16	0.40	258	5.17		
Leaders of your religious group in your community, if applicable	Strongly trust	450	11.38	450	9.00	22.21	<0.001
	Somewhat trust	832	21.04	984	19.69		
	Neither trust or distrust	1357	34.32	1888	37.78		
	Somewhat distrust	277	7.01	336	6.72		
	Strongly distrust	300	7.59	390	7.80		
	Not applicable	738	18.66	950	19.01		
The local elected officials who represent your community	Strongly trust	371	9.38	260	17.86	372.13	<0.001
	Somewhat trust	1289	32.60	1037	18.32		
	Neither trust or distrust	1441	36.44	1891	37.86		
	Somewhat distrust	549	13.88	915	20.76		
	Strongly distrust	304	7.69	892	5.21		
Social service, neighborhood, or civic organizations in your community	Strongly trust	294	5.89	519	13.14	551.31	<0.001
	Somewhat trust	1271	25.45	1549	39.23		
	Neither trust or distrust	2363	47.31	1558	39.45		
	Somewhat distrust	574	11.49	217	5.50		
	Strongly distrust	493	9.87	106	2.68		
Family and friends	Strongly trust	827	20.95	898	17.97	81.90	<0.001
	Somewhat trust	1643	41.62	1858	37.18		
	Neither trust or distrust	1226	31.05	1806	36.14		
	Somewhat distrust	183	4.64	223	4.46		
	Strongly distrust	69	1.75	212	4.24		

The final multivariable logistic regression model yielded several key findings; the results are summarized in Table 4. Trust in healthcare professionals remains a strong and significant predictor of COVID‐19 vaccine uptake. The strongest association was with trust in a personal doctor (AOR = 1.53, 95% confidence interval [CI]: 1.39–1.69, *p* < 0.001), indicating that for every one‐level increase on the trust scale, an individual's odds of being vaccinated increased by 53%. Significant positive associations were also found for trust in other doctors (AOR = 1.40, 95% CI: 1.27–1.54, *p* < 0.001), pharmacists (AOR = 1.32, 95% CI: 1.21–1.45, *p* < 0.001), social service, neighborhood, or civic organizations (AOR = 1.27, 95% CI: 1.18–1.37, *p* < 0.001), and local officials (AOR = 1.08, 95% CI: 1.01–1.15, *p* < 0.001). A critical finding is that trust in certain sources was associated with a lower likelihood of vaccine uptake. Higher trust in religious leaders was associated with a 19% decrease in the odds of COVID‐19 vaccine uptake (AOR = 0.81, 95% CI: 0.77–0.86, *p* < 0.001). Similarly, higher trust in family and friends was associated with a 15% decrease in the odds of COVID‐19 vaccine uptake (AOR = 0.85, 95% CI: 0.79–0.91, *p* < 0.001).

**TABLE 4:** Adjusted odds ratios from a multivariable logistic regression model predicting COVID‐19 vaccine uptake.

	Adjusted odds ratio (AOR)	95% confidence interval (CI)	*p*
**Trust in information sources (pre‐level increase)**			
Your personal doctor/Your personal healthcare provider/A doctor or healthcare provider in your community	1.53	1.39–1.69	<0.001
Other doctors or healthcare providers in your community	1.40	1.27–1.54	<0.001
Pharmacists in your community	1.32	1.21–1.45	<0.001
Leaders of your religious group in your community, if applicable	0.81	0.77–0.86	<0.001
The local elected officials who represent your community	1.08	1.01–1.15	<0.001
Social service, neighborhood, or civic organizations in your community	1.27	1.18–1.37	<0.001
Family and friends	0.85	0.79–0.91	<0.001
**Sociodemographic characteristics**			
Age (per year increase)	1.04	1.03–1.04	<0.001
Gender (ref. male)			
Female	1.03	0.92–1.14	0.635
Race/Ethnicity (ref. White, non‐Hispanic)			
Black, non‐Hispanic	0.96	0.81–1.14	0.663
Other, non‐Hispanic	1.13	0.87–1.45	0.355
2+ races, non‐Hispanic	1.06	0.79–1.42	0.709
Hispanic	1.31	1.14–1.52	<0.001
Education (ref. no. HS diploma/GED)			
High school graduate	1.92	1.63–2.25	<0.001
Some college/associate's	1.35	1.18–1.54	<0.001
Bachelor's or higher	0.97	0.87–1.08	0.554
General health (ref. poor)			
Fair	0.99	0.78–1.25	0.915
Good	0.85	0.69–1.04	0.108
Very good	0.95	0.81–1.11	0.499
Excellent	1.00	0.90–1.12	0.940

*Note:* Results are from the final parsimonious logistic regression model using imputed data. The model includes all trust variables and covariates simultaneously. For the trust variables and age, the AOR represents the odds for a one‐level increase on the 5‐point scale.

The multivariable logistic regression model also revealed that older age was consistently associated with a higher rate of COVID‐19 vaccine uptake (AOR = 1.04, 95% CI: 1.03–1.04, *p* < 0.001), with each additional year of age corresponding to a 4% increase in the odds of COVID‐19 vaccine uptake. Hispanic participants were significantly more likely than non‐Hispanic White participants to be vaccinated (AOR = 1.31, 95% CI: 1.14–1.52, *p* < 0.001). There were no other differences observed for non‐Hispanic Black, other non‐Hispanic, or multiracial participants compared with non‐Hispanic White participants. Education also played a role, as participants with a high school diploma (AOR = 1.92, 95% CI: 1.63–2.25, *p* < 0.001) and those with some college or an associate's degree (AOR = 1.35, 95% CI: 1.18–1.54, *p* < 0.001) had higher odds of vaccine uptake compared with those without a high school diploma/GED. However, having a bachelor's degree or higher was not significantly associated with vaccine uptake relative to those without a high school diploma (*p* = 0.55). Gender was not associated with vaccine uptake (*p* = 0.64). Self‐reported general health status was not significantly associated with COVID‐19 vaccine uptake. See Table 4 for details.

## 4 Discussion

The COVID‐19 pandemic underpinned the important role of trust in information sources in shaping health behaviors. This study explored how trust in specific information sources predicted COVID‐19 vaccine uptake. The results revealed a strong positive association between trust in healthcare professionals and COVID‐19 vaccine uptake. Participants who reported high trust in personal doctors, other doctors, or healthcare providers in the community and pharmacists were 1.53 to 1.32 times more likely to be vaccinated. Conversely, trust in non‐healthcare professionals, including elected officials and social service, neighborhood, or civic organizations, showed weaker associations, reflecting skepticism toward political or non‐health expert sources. In contrast, religious leaders and family/friends were among the least impactful information sources. Overall, the study findings on the role of trust in information sources and vaccine uptake are consistent with previous research highlighting the important influence of information sources. Individuals who trust official healthcare sources strongly predict vaccine uptake, which highlights the importance of credible information in shaping vaccination behaviors [19, 20]. Healthcare professionals significantly influence vaccination behaviors across diverse cultural contexts. In Europe, trust in healthcare workers outranked trust in political figures [21, 22], mirroring these US‐based results. However, in Nigeria, trust in healthcare workers significantly boosted vaccine uptake among rural populations [23]. The high degree of trust in healthcare providers observed in this study emphasized their potential to effectively counter vaccine hesitancy by serving as reliable sources of evidence‐based vaccine information. Additionally, these consistent cross‐cultural findings strongly support the principles of social cognitive theory (SCT), particularly observational learning, which suggests that individuals are likely to follow behaviors promoted by credible role models [24]. Because of their experience and close patient interactions, healthcare professionals serve as reliable “role models.” Healthcare professionals, with their extensive medical training, medical expertise, and personal patient interactions, are uniquely positioned to influence vaccine decisions. The strong positive association between trust in healthcare professionals and vaccine uptake emphasizes the importance of building and maintaining trust in the healthcare system. However, this trust reliance on healthcare professionals may not extend to diverse populations. Historical abuses and systemic inequities have fostered vaccine hesitancy and deep‐rooted distrust, posing ongoing challenges to trust in African American communities, weakening the role model effect of healthcare professionals [25, 26]. During the COVID‐19 pandemic, medical mistrust and low confidence in the healthcare system limited vaccine access and uptake among racial and ethnic minorities [16], compounded by language barriers, cultural differences, and perceptions of profit‐driven motives [26, 27]. These factors highlight the importance of addressing structural and systemic inequities and building trust to improve vaccine uptake in racial and ethnic populations. Mistrust of healthcare professionals can explain the mechanism that limits their influence on vaccine decisions, and efforts should be made to rebuild trust in healthcare professionals to improve vaccine uptake.

In contrast, trust in local elected officials and social service, neighborhood, or civic organizations showed less positive significant associations with vaccine uptake behavior. This reflects the non‐healthcare professionals’ limited credibility in public health crises. The lower level of trust in elected officials likely stems from the politicization of COVID‐19 in the United States, where partisan divides shaped public health perceptions [11]. Political rhetoric dismissing the virus's severity eroded confidence in government sources, reducing their influence on vaccine uptake behavior. Similarly, though positive, trust in social service, neighborhood, or civic organizations was less predictive. This result suggested that community‐level trust can also cultivate supportive environments that promote vaccine uptake through the reinforcement of positive social norms and the sharing of information [28]. Studies suggest that interactions with neighborhood and engagement with community‐based organizations can enhance vaccine acceptance by addressing local barriers and improving grassroots networks, particularly in underserved populations [3, 29]. However, this form of trust was less predictive of vaccine uptake compared to other factors, possibly due to community trust plays a complementary role in reinforcing behaviors rather than directly alleviating core concerns like vaccine safety or efficacy, especially amid widespread misinformation during the COVID‐19 pandemic [30]. These results underscore the need for integrated interventions that combine community engagement with efforts to build broader institutional trust for optimizing vaccine equity.

Interestingly, this study found that trust in religious leaders significantly negatively influences vaccine uptake. Given that health promotion programs involving faith‐based organizations and engaging faith leaders as advocates for disease prevention and treatment have been effective in improving community health [31], the findings of this study suggest that trust in religious leaders may not translate into greater vaccine uptake. It is crucial to explore how these organizations and leaders can be utilized to eliminate myths, clarify misunderstandings, build vaccine confidence, and encourage vaccination. This could also be a reflection of cultural differences. Although religious leaders have significant communal authority in Nigeria, their influence over health decisions may be limited [32]. In contrast, secular trends and the separation of church and state in the United States further diminish such influence. Our sample's large degree of variation in trust in religious leaders (37.78%–6.72%) points to a range of opinions that may be influenced by political or denominational considerations. This cultural complexity deserves more investigation in a variety of contexts.

Trust in family/friends negatively impacted the COVID‐19 vaccine uptake. This may be due to the pervasive spread of misinformation within personal and social networks. Exposure to vaccine‐related misinformation within social circles, particularly among family and friends, can significantly decrease an individual's intention for vaccine uptake [33]. The low level of trust in family and friends can also negatively affect vaccination rates among children and adolescents [34]. When parents with young children and adolescents receive conflicting information or do not trust their adults and friends, they may feel confused and ultimately may not get their children vaccinated [34]. This concern can be addressed by encouraging healthcare providers to share evidence‐based information and personal experiences about the importance of vaccines. Additionally, organizing community events can be used to educate families about vaccines, which can help build trust and improve vaccine uptake. These dynamics suggest that non‐healthcare sources are a less reliable mechanism for health information in polarized or misinformed environments.

Sociodemographic variables also demonstrated significant implications. Aligning with global trends, older participants showed greater vaccine uptake, indicating these groups are less susceptible to misinformation and trust the healthcare providers [35]. This study finding also revealed that educational attainment may play a role in COVID‐19 vaccine uptake, but the effect was not linear across levels of education. One possible explanation is that individuals with lower educational attainment may benefit more from targeted public health campaigns and community‐based efforts aimed at increasing awareness and accessibility [36]. In contrast, those with higher education may also be more exposed to vaccine‐related misinformation, skepticism, or structural barriers unrelated to education itself, potentially neutralizing the expected positive effect. Similar nonlinear associations also have been reported in prior studies, indicating that higher education alone does not necessarily guarantee greater vaccine uptake [37, 38].

Perceived general health status was not significantly associated with COVID‐19 vaccine uptake. This suggested that perceived health status alone may not drive vaccine decision‐making. Instead, other factors, such as perceived susceptibility to COVID‐19, trust in health information sources, or structural barriers to vaccination, may be more influential than self‐rated health in shaping vaccine behavior. These findings echoed with studies showing that health perceptions are less predictive of vaccination than sociodemographic factors or trust‐related variables [39, 40]. In addition, the time of data collection during the COVID‐19 pandemic may further explain these results, as shaped public health messaging, urgency, and vaccine availability during the period likely enlarged the influence of other factors, such as trust, than individual health perceptions.

Although the bivariate analysis results of this study suggested significant differences across racial and ethnic groups, only Hispanic participants remained significantly more likely when compared with non‐Hispanic White participants to be vaccinated in the adjusted model. In contrast, no significant differences in vaccination uptake were observed for non‐Hispanic Black, other non‐Hispanic, or multiracial participants compared to non‐Hispanic White participants. The positive vaccination uptake trends among Hispanic participants could reflect effective culturally tailored outreach efforts and community‐based initiatives [36]. These efforts were more likely to improve the linguistic accessibility, community trust, and targeted messaging to address barriers specific to Hispanic populations [20]. Although vaccine hesitancy may persist among the African American populations due to historical distrust from events like the Tuskegee Syphilis Study, these findings also demonstrated that outreach and engagement strategies may not have fully addressed the concerns or structural barriers experienced by these groups [41]. Among the Indigenous Native Hawaiian and Pacific Islander populations, Juarez et al. [42] found that trust in unofficial healthcare sources was associated with greater vaccine hesitancy, highlighting how reliance on nonofficial healthcare information can negatively influence vaccine uptake behavior. Similarly, this study's findings indicate that the influence of trust varies depending on both the information sources and the sociocultural context, emphasizing the importance of culturally tailored interventions that not only build trust but also promote evidence‐based information sources. Addressing these disparities requires nuanced interventions that integrate trust‐building and practical accessibility measures to enhance vaccine equity, such as the CDC's Partnering for Vaccine Equity (P4VE) program. These approaches can ensure sustained progress in addressing disparities and promoting equitable vaccine uptake.

### 4.1 Implications

Based on the study's findings that trust in information sources significantly influences COVID‐19 vaccine uptake, the following implementation strategies are recommended to enhance public health interventions. At the individual level, strengthening healthcare provider–patient communication through targeted training to enhance patient trust and self‐efficacy regarding vaccination. Additionally, appointing trustworthy community members as vaccination ambassadors can help model positive behaviors and reinforce social norms, enabling communities to make informed health decisions.

Public health campaigns should adopt a community‐centric approach by improving trusted non‐health professional channels to maximize the reach and credibility of health messages. Specifically, partnerships with local leaders, faith‐based groups, and culturally relevant social media influencers should be strengthened by engaging with them and providing them with scientific evidence to effectively disseminate accurate vaccine information and counter disinformation, which has been a major obstacle to vaccine acceptance. Furthermore, engaging local elected officials to promote these messages can further amplify community involvement, dispel myths, and ensure factual information reaches target audiences.

Complementary interventions are essential to sustain these efforts. Tackling health literacy disparities requires tailored communication strategies, such as developing visual materials for low‐literacy populations and launching proactive school‐based programs to equip younger generations with critical thinking skills against misinformation. To address misinformation directly, regulations on digital platforms to limit the spread of false content while prioritizing scientifically accurate information may help.

### 4.2 Limitations

There are several limitations to this study that should be taken into account. First, the HRMS is an online, opt‐in survey that may introduce self‐selection bias. Participants who choose to respond were more likely to be digitally literate, health‐conscious, and motivated, which may potentially inflate reported levels of trust in healthcare providers and vaccine uptake. This method of sampling also limits representativeness, underrepresenting vulnerable groups, such as those of low socioeconomic status, rural populations, and immigrants. These populations often face unique trust dynamics, higher vaccine hesitancy, and structural barriers, such as limited healthcare access. This underrepresentation constrains the generalizability of the study findings, especially for understanding trust in offline networks, such as religious leaders, family/friends, or other social networks, that significantly influence vaccine behavior among these groups. Second, self‐reported data may introduce social desirability bias, particularly in politically polarized contexts where respondents may overstate positive behaviors or attitudes to align with perceived norms, which could exaggerate the observed associations between trust in information sources and vaccine uptake. Third, the cross‐sectional design, with data collected in April 2021 during the early phase of the COVID‐19 vaccine rollout, limited our ability to establish causality between trust in information sources and vaccine uptake. At this time, vaccine availability and eligibility varied widely, meaning uptake may reflect access constraints rather than trust alone. This timing also restricted insights into how trust evolved in response to new COVID‐19 variants, policy changes, or shifting public perceptions. Finally, the US sample restricted generalizability, especially to low‐income countries, where mistrust of international health organizations may exhibit unique trends, for example, due to unfair vaccine distribution or colonial medical abuses in the past.

Future studies should fill these gaps by employing more diverse sampling strategies, such as community‐based recruitment to include underrepresented groups, using experimental designs to compare the effectiveness of trust‐building interventions, such as community leader engagement versus digital influencer campaigns, and applying longitudinal designs to investigate how trust changes in the face of new COVID‐19 variants or policy changes. Qualitative studies could also provide deeper insights into the more nuanced relationships between trust in information sources, historical factors, and vaccine uptake in diverse populations. The relationship between past trauma and current faith in healthcare systems should also be investigated further because these factors may significantly influence vaccine uptake behavior in underserved groups globally.

## 5 Conclusion

Trust is not merely a facilitator but a prerequisite for effective health crisis response. As variants evolve and booster campaigns intensify, public health efforts must prioritize trust‐building through culturally resonant, equity‐focused strategies. By integrating multi‐level, theory‐driven interventions, which include community engagement, policy reform, and educational outreach, public health initiatives can harness trust to boost vaccine uptake, enhance resilience against disinformation, and foster healthier, more informed communities. In addition, by centering healthcare providers, empowering communities, and confronting misinformation at its roots, global health systems can transform vaccine hesitancy into confidence, ensuring that trust, not fear, guides the path to recovery.

## Author Contributions

**Peijia Zha:** conceptualization, investigation, writing – original draft, methodology, writing – review and editing, formal analysis. **Rubab Qureshi:** conceptualization, writing – original draft, methodology, writing – review and editing. **Ganga Mahat:** writing – original draft, writing – review and editing. **Le Gao:** methodology, software, formal analysis, writing – review and editing. **Catherine Garcia:** conceptualization, writing – original draft, project administration. **Zhi Wei:** methodology, validation, formal analysis, data curation, writing – review and editing.

## Funding

The authors have nothing to report

## Ethics Statement

This study utilized a publicly available dataset obtained from the Inter‐University Consortium for Political and Social Research and the Regents of the University of Michigan (https://www.icpsr.umich.edu). Ethical approval for this analysis was waived because the data were not collected by the authors and are publicly accessible, with no further collection of human data involved. The data are available at https://www.icpsr.umich.edu/web/ICPSR/studies/38526. The authors confirm that the research presented in this manuscript met the ethical guidelines.

## Conflicts of Interest

The authors declare no conflicts of interest.

## Data Availability

The data used in this study are publicly available and were downloaded from the Inter‐University Consortium for Political and Social Research and the Regents of the University of Michigan (https://www.icpsr.umich.edu). The dataset is accessible at https://www.icpsr.umich.edu/web/ICPSR/studies/38526.
